# A splice site mutation in *CRYBA1/A3* causing autosomal dominant posterior polar cataract in a Chinese pedigree

**Published:** 2010-02-05

**Authors:** Zhensheng Gu, Baohu Ji, Chunling Wan, Guang He, Juan Zhang, Ming Zhang, Guoyin Feng, Lin He, Linghan Gao

**Affiliations:** 1 Department of Ophthalmology, Xinhua Hospital, Medical College of Shanghai Jiao Tong University, Shanghai, China; 2 Bio-X Center, Key Laboratory for the Genetics of Developmental and Neuropsychiatric Disorders (Ministry of Education), Shanghai Jiao Tong University, Shanghai, China; 3 Institute for Nutritional Sciences, Shanghai Institute of Biological Sciences, Chinese Academy of Sciences, Shanghai, China; 4 Institutes of Biomedical Sciences, Fudan University, Shanghai, China

## Abstract

**Purpose:**

To identify the mutant gene for autosomal dominant posterior polar congenital cataract in a four-generation Chinese pedigree.

**Methods:**

The clinical data of patients from the family were recorded by slit-lamp photography. Genomic DNA samples from peripheral blood of the pedigree members were then isolated to map the relevant gene, using microsatellite markers for two-point linkage analysis. Genotype and haplotypes of the pedigree were constructed using Cyrillic software to locate the relevant region. Direct sequencing was performed to screen out the disease-causing mutation.

**Results:**

The congenital cataract phenotype of the pedigree was labeled as the posterior polar type by using slit-lamp photography. Linkage analysis results indicated a maximum logarithm of odds LOD score of (*Z*_max_) 2.02 at D17S1800 (θ_max_=0.00). Haplotyping identified a 26-cM region flanked by D17S921 and D17S800 on 17p12–21.2, namely at the βA1/A3-crystallin (*CRYBA1/A3*) gene locus. Sequencing revealed a splice site mutation, G→A, at the first base of intron 3 of *CRYBA1/A3*, which co-segregated with the affected individuals in the pedigree but which was not found in the unaffected members of the family or in the 50 unrelated controls.

**Conclusions:**

Our results demonstrated that a splice site mutation of *CRYBA1/A3* was responsible for the autosomal dominant posterior polar congenital cataract in a four-generation Chinese pedigree. The same mutation in this gene had previously been reported to be associated with other phenotype cataracts. This study is the first report relating a mutation of *CRYBA1/A3* to posterior polar cataract.

## Introduction

Congenital cataract (CC) refers to lens opacification, which results in visual impairment or blindness at birth or in early childhood and accounts for one-tenth of childhood blindness throughout the world. Clinically, according to morphology, such as the outward appearance, size, and location of lens opacity, congenital cataract can be classified into several subtypes: whole lens, nuclear, lamellar, cortical, polar, sutural, pulverulent, cerulean, coralliform, and other minor subtypes [1]. Congenital cataract is usually present as an isolated trait, part of systemic syndromes, or concomitant with other ocular anomalies. Genetically, the majority of isolated congenital cataracts exhibit as autosomal dominant, although autosomal recessive and X-linked inherited forms have also been reported. As a heterogeneous disorder, whether as a clinical or genetic phenomenon, congenital cataract has puzzled researchers for some time.

Recent evidence suggests that a genetic factor plays an important role in the pathogenesis of congenital cataract. More than 20 genes have been identified to be associated with isolated congenital cataract, encoding proteins, such as crystallins, the major structural lens proteins (*CRYAA*, *CRYAB*, *CRYBA1/A3*, *CRYBB1*, *CRYBB2*, *CRYBB3*, *CRYGC*, *CRYGD*, and *CRYGS*), gap junctional proteins (*GJA3* and *GJA8*), major intrinsic protein (*MIP*/*MIP26*), lens intrinsic membrane protein 2 (*LIM2*/*MP19*), beaded filament proteins (*BFSP1*, *BFSP2*), heat shock protein (*HSF4*), paired-like homeodomain transcription factor 3 (*PITX3*) galactokinase transferase 2 (*GCNT2*), transmembrane protein 114 (*TMEM114*), and v-maf musculoaponeurotic fibrosarcoma oncogene homologue (*MAF*). Several other loci have also been linked to congenital cataract, but the mutant gene(s) has, so far, remained elusive [2].

In this study we report a Chinese four-generation pedigree with autosomal dominant posterior polar congenital cataract. A splice mutation, G→A transition of the 5′ splice site of intron 3, was indentified in *CRYBA1/A3* in all the pedigree patients. This is the first report to relate a mutation of *CRYBA1/A3* to posterior polar cataract.

## Methods

### Clinical examinations

A four-generation Chinese pedigree with cataract was recruited from Jiangsu province in China for this study. Full family medical history was recorded by interviewing the family members. All participating members underwent careful ophthalmic examination of the relevant optical features, including visual acuity tests and slit-lamp and fundus examination of the dilated pupil. Slit-lamp photography was performed to record the cataract phenotype of the patients. Fifty unrelated subjects without cataracts were recruited from the Ophthalmology Clinic of Xinhua Hospital (Shanghai, China) as normal controls. The research was approved by the ethics committee of Xinhua Hospital. Informed consent was signed in accordance with the Helsinki Declaration by the members who participated in this study.

### Microsatellite genotyping

About 2 ml of peripheral blood was drawn from the members who took part in the study. The blood was then mixed with trisodium citrate and stored at 0 °C. Genomic DNA was extracted from peripheral blood using the QIAamp DNA kit Blood Mini (Qiagen, Santa Clara, CA). Genes known to be related to congenital cataract were chosen for GeneScan and linkage analysis. The whole process of GeneScan is below.

A three-temperature touchdown PCR program was performed for DNA amplifying: 96 °C for 12 min; followed by 14 cycles at 95 °C for 30 s, 64 °C for 30 s (with temperature decreasing from 64 °C to 57 °C by −0.5 °C per cycle), 72 °C for 1 min; followed by 30 cycles at 95 °C for 30 s, 57 °C for 30 s, and 72 °C for 1 min; and a final extension at 72 °C for 10 min, using the Gene Amp PCR System 9700 (Applied Biosystems, Foster City, CA).

A standard PCR reaction was performed in a 5-μl volume containing 20 ng genomic DNA, 0.1 μM of each primer, 300 μM dNTP, 1 µl of 10× PCR buffer, 7 mM MgCl_2_, and 0.3 U Hotstart Taq (Qiagen).

Candidate gene screening for linkage analysis was performed using microsatellite markers (fluorescent-labeled primers) based on NCBI. The PCR products were mixed according to size (Genescan-400HD ROX; PE Applied Biosystems), denatured at 95 °C for 1 min, and electrophoresed on a 96-capillary automated DNA sequencer (MegaBACE 1000; Amersham, Freiburg, Germany). The results were analyzed on Genetic Profiler software (version 1.5; Amersham).

### Linkage analysis and haplotyping

Two-point linkage analysis was calculated using the MLINK subprogram from the LINKAGE package (version 5.1, Rockefeller University, New York, NY). A gene frequency of 0.0001 and penetrance of 100% were assumed. Microsatellite markers, allele frequencies, and recombination distances between the marker loci were based on the Marshfield database and the UCSC database. Family and haplotype data were processed using Cyrillic software (version 2.1, Cyrillic, Oxfordshire, UK).

### Gene sequencing

All coding exons, splice regions, and UTRs were amplified using designed *CRYBA1/A3* primers. Sequencing reactions were performed on both strands, using the BigDye Terminator Cycle Sequencing Kit v3.1 (Applied Biosystems) on an ABI PRISMTM 3100 analyzer. Sequencing results were analyzed using Sequence Scanner v1.0 software (Applied Biosystems), referencing the NCBI GeneBank (NM_005208 for *CRYBA1/A3*). Mutation was confirmed in all family subjects and controls.

## Results

### Clinical features

**Figure 1:**
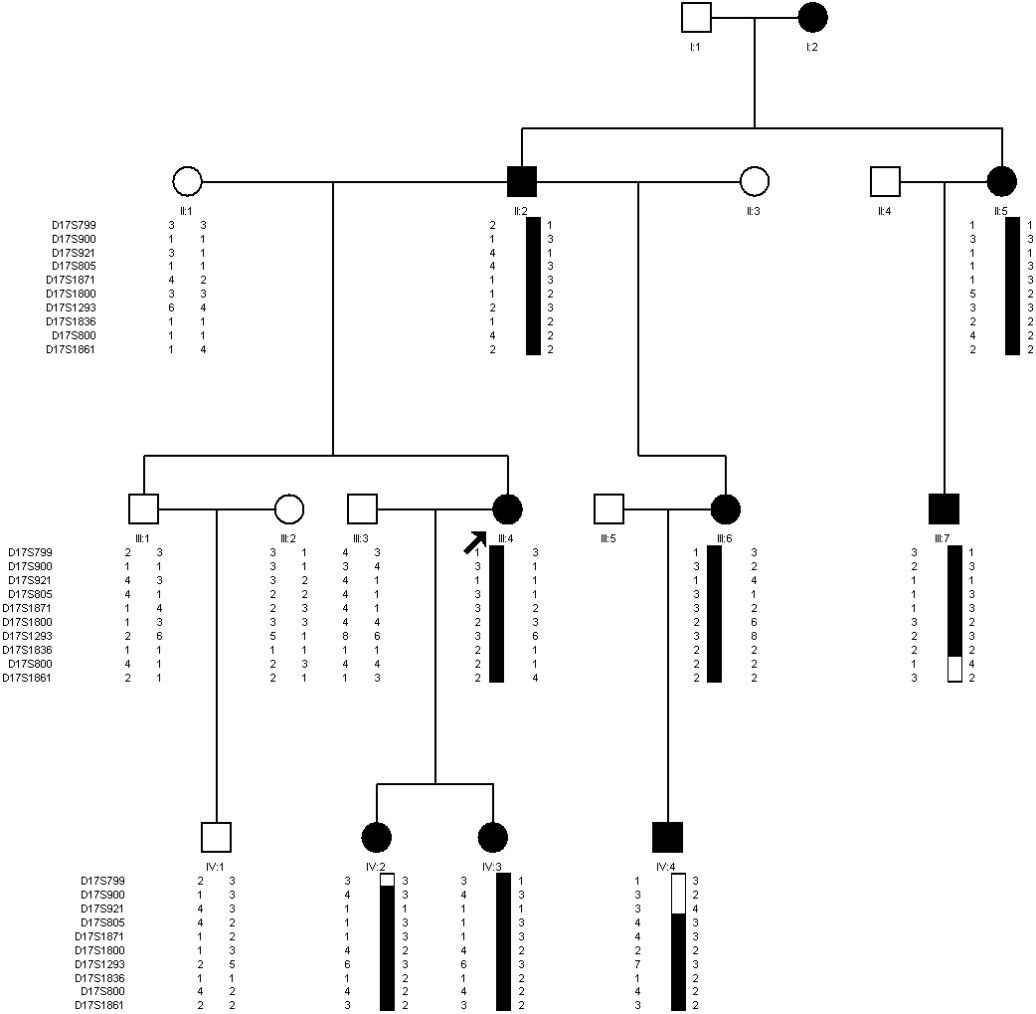
Pedigree and haplotypes of the posterior polar cataract family. Square and circles symbols denote males and females respectively; affected individuals are shaded black; the proband is identified by an arrow. Haplotyping markers are shown at the left of each generation. Black and white bars depict the disease and non-disease associated haplotype respectively. Haplotype analysis defined the causative gene as being between D17S921 and D17S800 on 17p12-21.2.

**Figure 2:**
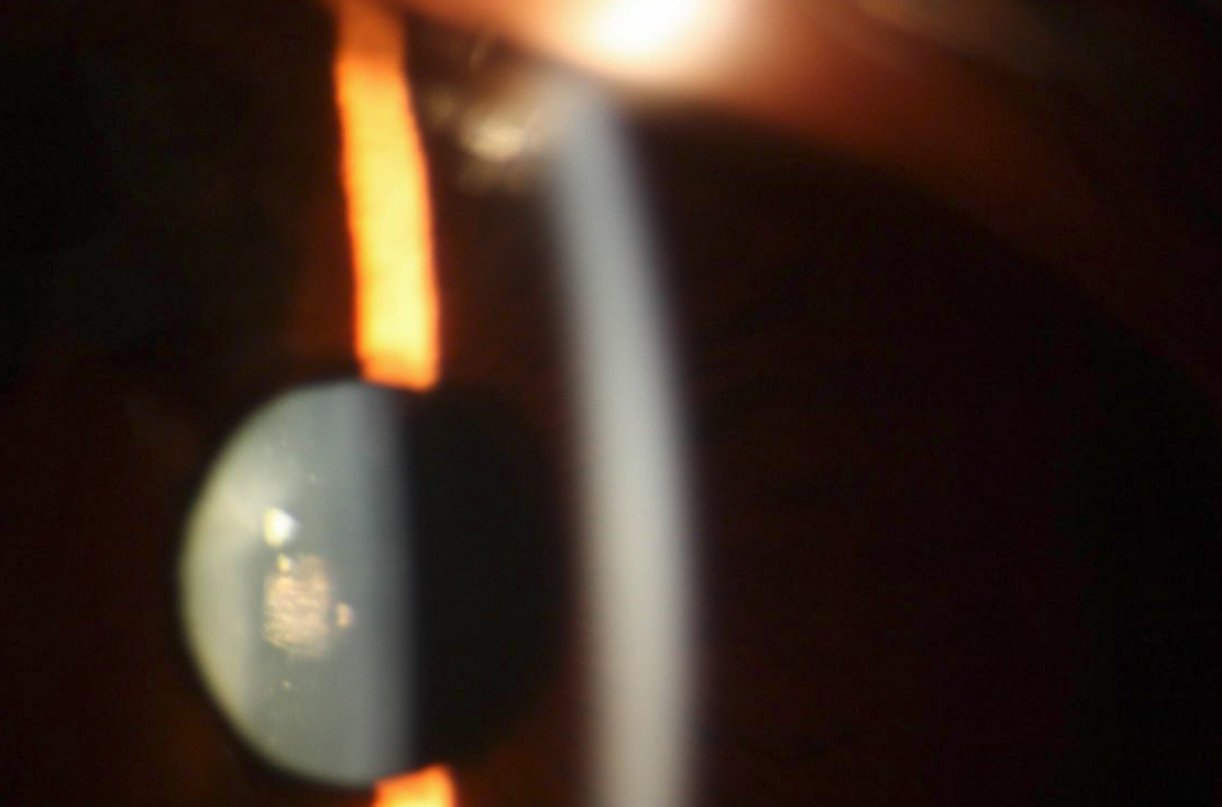
Slit-lamp photograph of the posterior polar cataract taken from patient IV:4 at 25 years of age.

A four-generation Chinese pedigree that consists of 18 individuals, including nine affected individuals, provided the basis for the study. Thirteen family members participated in the study (eight affected and five unaffected individuals; Figure 1). All patients in this pedigree had bilateral cataract and had shown symptoms of vision decrease since their early teens. Slit-lamp examination revealed opacification consisting of a dense gray-white deposit that affected the posterior polar or posterior cortex of the lens (Figure 2). There was no history of other ocular or systemic abnormalities in the family. The proband (III:4) had been diagnosed with bilateral cataract before the age of 10. She was unable to undergo cataract extraction for economic reasons. Her best corrected visual acuity in a lit room was only 20/200 in both eyes. The two daughters of the proband (IV:2 and IV:3) were both diagnosed as having cataract and had mild visual loss at the age of 3. Lens opacity progressively worsened as they grew older. They underwent cataract removal and intraocular lens implantation before reaching school age, but unfortunately, about 1 year later, they had to undergo Nd:YAG Laser treatment for posterior capsular opacification. Apart from the two daughters of the proband, no patients in this family had undergone cataract surgery. In terms of lens opacity the range of best corrected visual acuity for the family was between 20/200 and 10/30. Medical records indicated that cataract affected four successive generations, which suggests the dominant mode. Male–male transmission was not present in the family, and the transmission patterns of the pedigree were therefore consistent with autosomal dominant or X-link dominant mode.

### Linkage and haplotype analysis

Before undertaking the study we had already made linkage calculations for the family based on assumed data. The data were assumed along such rules: the total genotypes of a certain marker were five kinds (1, 2, 3, 4, and 5); a certain genotype (1) was only shared by all patients. The estimated logarithm of odds LOD score was just above 2 on the autosomal dominant mode, given the limited size of the family. Then, genescan and linkage analysis were first performed in the known loci for posterior polar cataract (1p36, 10q25, 11q22-q23, 14q22-q23, 16q22, and 20p12-q12), but negative LOD scores were obtained (data not shown). Thus, subsequent analysis embraced the known loci of congenital cataract, and an indicative result was obtained on chromosome 17 at D17S1871 (Z=1.75, θ=0.00). Additional markers were added for further study, and more positive results were obtained (Table 1), including the maximum LOD score (Z_max_) 2.02 at D17S1800 (θ_max_=0.00), which confirmed this region and excluded an X-link inheritance mode in the pedigree. Haplotypes were constructed using the above mentioned markers. Three informative recombination events were observed in the pedigree. Recombination events occurred in individual IV:2 and IV:4, indicating the distal boundary of the region to be between D17S921 and D17S805. A third recombination event occurred in individual III:7, suggesting that the proximal border was between marker D17S1836 and D17S800. Thus, the affected individuals in this family shared a defined 26-cM region flanked by markers D17S921 and D17S800 (Figure 1) on chromosome 17p12-q21.2, which was responsible for disease.

**Table 1:** Two point LOD scores for autosomal dominant posterior polar cataract on chromosome 17p.

**Marker**	**CM**	**LOD score at *θ* =**						
		**0.0**	**0.1**	**0.2**	**0.3**	**0.4**	***Z*_max_**	***θ*_max_**
D17S799	31.96	−2.67	0.19	0.22	0.13	0.03	0.22	0.2
D17S900	36.14	1.45	1.15	0.83	0.50	0.21	1.45	0.0
D17S921	36.14	−3.15	−0.06	0.04	0.04	0.01	0.04	0.2
D17S805	47.00	1.72	1.37	1.01	0.64	0.29	1.72	0.0
D17S1871	48.07	1.75	1.40	1.03	0.65	0.30	1.75	0.0
D17S1800	51.63	2.02	1.62	1.20	0.75	0.33	2.02	0.0
D17S1293	56.48	1.75	1.40	1.03	0.65	0.29	1.75	0.0
D17S1836	60.40	1.45	1.15	0.83	0.50	0.21	1.45	0.0
D17S800	62.01	−2.41	0.46	0.45	0.33	0.17	0.46	0.1
D17S1861	63.62	0.85	0.68	0.51	0.34	0.17	0.85	0.0

### Mutation analysis for *CRYBA1/A3*

**Figure 3:**
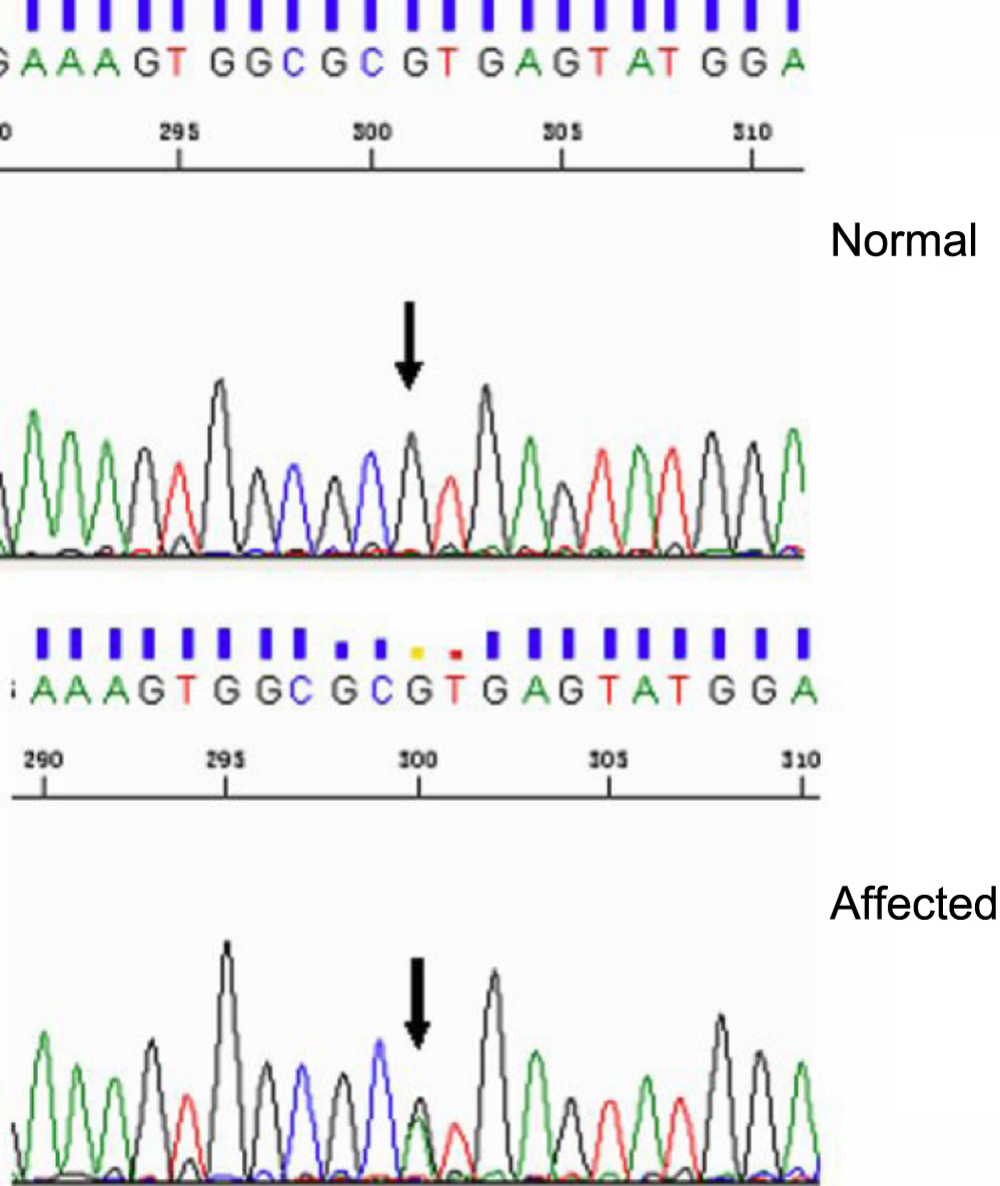
Sequence analysis of the Chinese pedigree with autosomal dominant posterior polar cataract. A splice site mutation at the first base of intron 3 (G→A) , which is also  identified at position 474 in the donor splice junction of intron 3 was co-segrated with all patients in the family, but was not found in the unaffected family members  nor in the 50 unrelated control subjects.

The most significant candidate gene for congenital cataract, *CRYBA1/A3*, which is located just at the identified locus, was sequenced. Direct sequencing of the exons, exon/intron boundries, and UTRs were performed in two affected and two unaffected individuals of the pedigree. Sequencing in *CRYBA1/A3* revealed a splice site mutation at the first base of intron 3 (G→A), that is, at position 474 in the donor splice junction of intron 3 (Figure 3). The mutation was also found in all family patients but not in the unaffected family members nor in the 50 unrelated control subjects. Thus, the splice site mutation was identified to co-segregate with posterior polar cataract in this pedigree.

## Discussion

In this study we identified a splice site mutation of *CRYBA1/A3* in a four-generation Chinese pedigree with autosomal dominant posterior polar cataract. Posterior polar cataracts (CTPP) refer to subcapsular opacities located at the back of the lens and may impair visual acuity because of proximity to the optical center of the eye. Usually posterior lens opacities can be triggered by senility, diabetes, or steroidal treatment or can accompany certain syndromes, such as neurofibromatosis type II or retinitis pigmentosa. Familial posterior polar cataract is genetically heterogeneous and often inherited as an autosomal dominant trait. To date, four genes for posterior polar cataract have been identified. CTPP1 (OMIM 116600) has been mapped to 1p36 [3]. Mutations of the *EPHA2* gene in this region have been found to be associated with congenital cataract in different families, including G948W [4], T940I, c.2915_2916delTG, and c.2826–9G>A [5]. CTPP2 has been associated with *CRYAB* on 11q22-q22.3, and a Pro20Ser mutation and a deletion mutation (450delA) have also been highlighted [6,7]. The *CHMP4B* gene on chromosome 20p12-q12 is responsible for CTPP3 (OMIM 605387). Further study revealed a D129V mutation co-segregated with progressive, childhood, posterior, subcapsular cataract in a Caucasian family, and an E161K mutation has been identified as being associated with posterior polar cataract in a Japanese family [8]. Three mutations of *PITX3* gene on chromosome 10q25, 38G>A mutation, 17-bp insertion, and 650delG have been reported to cause CTPP4 (OMIM 610623) [9-12]. Two loci with unknown genes have similarly been reported, 14q22-q23 for CTPP5 (OMIM 610634) [13] and 16q22 [14].

As abundantly expressed proteins (accounting for approximately 90% of the total protein) in the lens, water-soluble crystallins play an important role in maintaining lens transparency as they are expressed in the lens from the beginning of its development. Mutant crystallin may alter stability, solubility, or ability to oligomerize and is likely to precipitate from solution, resulting in lens opacity. They are, therefore, considered to be good candidate genes for congenital cataract [2,15]. The crystallins are divided into α-, β-, and γ-crystallin groups according to decreasing molecular weight of the native protein. The β- and γ-crystallins are considered to belong to the same super family with highly similar core sequences evolving from the same ancestral gene. A common polypeptide chain fold shared by the β- and γ-crystallins has been entitled Greek key motif and comprises four antiparallel β-sheets. Although the function of the Greek key motif is still unclear, computer-based analysis suggests that it forms an interdomain association—intermolecular in the β-crystallins and intramolecular in the γ-crystallins [16]. The β-crystallins are expressed at a high level in the lens and form into dimers, tetramers, and higher order complexes to maintain lens transparency and refractivity. The β-crystallins embrace more acidic (βA-) crystallins, which are encoded by four genes (*CRYBA1*, *CRYBA2*, *CRYBA3*, and *CRYBA4*), and more basic (βB-) crystallins, which are encoded by three genes (*CRYBB1*, *CRYBB2*, and *CRYBB3*) [17]. The β-crystallins are major protein components in the human lens. They can interact with each other or with other lens proteins so that their stability and association into higher order complexes are critical for lens clarity and refraction. Dimerization of β-crystallins is energetically highly favored and rapidly reversible under certain physiologic conditions. Disruption of stabilization or oligomerization would result in lens opacification.

*CRYBA1* (also called *CRYBA1/A3*) comprises six exons that encode two proteins (A1- and βA3-crystallins) by using an alternative translation initiation site. The βA1- and βA3-crystallins consist of seven protein regions: four homologous (Greek key) motifs, a connecting peptide, and NH_2_- and COOH-terminal extensions. These two proteins are identical except for 17 additional amino acid residues at the NH_2_-terminal arm of the βA3-crystallin. The βA1/A3-crystallin has been suggested as favoring other more stable crystallin subunit interactions [18]. Study in vitro has also demonstrated that βB1- and βA3-crystallin would form a heterocomplex under certain physiologic conditions [4]. Two SNPs of *CRYBA1/A3*, which lead to amino acid exchanges, were found: rs1129656, the A/T nucleotide transversion results in an Ile116Phe substitution; and rs1129658, the C/T nucleotide transition results in a Pro174Leu substitution. Two major mutations of *CRYBA1/A3* have recently been reported as being associated with congenital cataract in different families. One is a 3-bp (GGA) deletion at nucleotide position 276–281, which causes an in-frame deletion of a glycine residue at position 91 (ΔG91). This mutation was also reported in an English family with lamellar cataract [19], in a Chinese family with nuclear cataract [20], in a Swiss family with suture-sparing nuclear cataract [21], and in two Chinese families with pulverulent nuclear cataracts and pulverulent lamellar cataract [22]. The other mutation is the splice site mutation, which has also been associated with various cataract phenotypes in several families. The first base of the 5′ donor splice site of intron 3 (nucleotide position 24601445) is the mutant hotspot. The G→A transition of this base was observed in an Indian family with zonular sutural cataracts [15], in an Australian family with nucleus and Y-sutural opacities [23], and in an Indian family with zonular lamellar opacification [24]. The G→C transition in an identical locus was found in a Brazilian family presenting pulverulent cataract [25]. The G at position +1 of the 5′ (donor) splice site is highly conserved so that mutation of this base can be expected to skip the donor splice junction, or to recruit a cryptic splice site, or possibly a combination of both. Skipping of the splicing site may mistakenly join exons of *CRYBA1/A3* and lead to premature termination, which can damage protein and finally cause cataract [15].

In the present study we identified a previously reported mutation, G→A transition of a 5′ splice site of intron 3, in a Chinese pedigree with posterior polar cataract. It is the first report to relate a mutation of *CRYBA1/A3* to posterior polar cataract. This study provides more evidence of genetic heterogeneity of congenital cataract and may help to further clarify the pathogenesis of cataract.
